# Pterostilbene in the Management and Treatment of Multiple Myeloma

**DOI:** 10.3390/cimb48020216

**Published:** 2026-02-16

**Authors:** Benjamin S. Buehrer, Adam R. Grden, Ethan Johnson, Manav Y. Patel, Rupesh Raina

**Affiliations:** 1 College of Medicine, Northeast Ohio Medical University, Rootstown, OH 44272, USA; bbuehrer@neomed.edu (B.S.B.); agrden@neomed.edu (A.R.G.); ejohnson4@neomed.edu (E.J.); mpatel22@neomed.edu (M.Y.P.); 2 Department of Pediatric Nephrology, Akron Children’s Hospital, Akron, OH 44308, USA; 3 Department of Nephrology, Akron Nephrology Associates/Cleveland Clinic Akron General Medical Center, Akron, OH 44302, USA

**Keywords:** hematology, oncology, antioxidant, chemotherapy, multiple myeloma

## Abstract

Multiple Myeloma is a malignancy of the plasma cells of the bone marrow. This cancer rapidly becomes refractory to many of the currently available chemotherapeutic regimens used against it, requiring an alternative option or supplementary therapy. Pterostilbene is a naturally occurring compound in a variety of commonly consumed plants that exhibits strong antioxidant properties and, lately, has shown increasing activity as an anti-neoplastic compound. A review of the literature published since 2015 on Google Scholar and PubMed was conducted, with a focus on randomized controlled trials and an exclusion of review articles, unless pertinent to pathophysiology of Multiple Myeloma or background on Pterostilbene as a compound. Though data is limited in the use of Pterostilbene as an agent to combat Multiple Myeloma, studies have shown that it, along with synthetic derivatives, can induce apoptosis and limit proliferation of Multiple Myeloma cell lines. While safety has been evaluated in several settings with promising results, for Pterostilbene to be considered as a supplementary treatment in Multiple Myeloma, safe and effective doses of the compound in this patient population must be investigated and established via pre-clinical and clinical trials in the future.

## 1. Introduction

Pterostilbene (trans-3,5-dimethoxy-4-hydroxystilbene) (PT) is a natural compound that is the primary antioxidant found in blueberries. The compound is found at levels from 99 to 520 ng/g of dry weight in various berries in the Vaccinium genus [1]. PT is also found in several varieties of grapes [2]. The compound is a highly bioavailable analog of resveratrol, a well-studied compound which, along with PT, is part of the Stilbene group of phenolic compounds. PT was found to have 80–95% bioavailability when administered orally in rats, as compared to only 20% for resveratrol [2,3]. Extensive studies have shown potential therapeutic and protective effects of PT via its antioxidant and anti-inflammatory properties. Additionally, previous studies have shown antineoplastic mechanisms, which may represent potential therapeutic uses in the treatment or prevention of cancers.

Multiple Myeloma (MM) is a cancer characterized by monoclonal hyperplasia of the plasma cells of the bone marrow, often arising from monoclonal gammopathy of undetermined significance (MGUS), a generally benign plasma cell disorder. MM is the second most common hematologic malignancy and is most common in older adults, with a median age at diagnosis of 65 years [4]. The overgrowth of plasma cells seen in MM leads to an overproduction and accumulation of nonfunctional immunoglobulins in the patient. These faulty immunoglobulins lead to various issues including bone lesions, anemia, infections, hypercalcemia, renal failure, and an immunocompromised state. The pathogenesis of this disease has been researched extensively and has yielded potential targets for immunotherapy in MM. There is currently no known cure for MM, but as the mean survival of a sample of 1000 patients treated with lenalidomide + bortezomib + dexamethasone between 2007 and 2016 was found to be 126.6 months from diagnosis, there are many opportunities to prolong survival times with a supplemental or alternative treatment [5].

For this narrative review, a literature search of Google Scholar and PubMed was conducted using the search terms “pterostilbene”, “cancer”, and “multiple myeloma”, including only publications since 2015 to ensure the most recent data was the focus of this article. Randomized controlled trials conducted in vitro and in vivo were primarily used due to their value in providing mechanistic data. All review articles were excluded, unless used for background information. For data extraction, key findings were collected and organized by mechanism of cancer inhibition in different types of cancers. These findings were synthesized in the discussion and used to describe PT’s effect on MM cell lines specifically. Zotero was utilized to organize and cite all articles used in this review.

Currently, limited research exists regarding the use of PT in the treatment of MM. PT has been found to induce apoptosis and inhibit growth of MM cells via multiple pathways in vitro and in vivo [6,7]. A handful of novel compounds derived from PT have been synthesized by research teams investigating their effects on MM cell lines [8,9,10,11]. Of the three compounds found to be tested against MM cell lines, all showed promise both in vitro and in vivo. Gaps exist when it comes to determining how these novel compounds would fare in clinical trials in terms of both safety and efficacy, and if they could be viable options as adjunctive treatments for MM to extend survival times. This review aims to summarize the anti-cancer properties of PT and how those properties could specifically combat the mechanisms by which MM cells proliferate in the human body.

## 2. Discussion

### 2.1. Chemical and Biological Properties of Pterostilbene

PT is a naturally occurring compound that is a dimethyl ether analog of the highly-researched compound resveratrol. These compounds belong to a class of naturally occurring chemicals known as polyphenols, which are found in various plants and their fruits. Since resveratrol was attributed to being responsible for the cardioprotective effects found from red wine consumption in the 1990s, it and other polyphenols became the subjects of a host of studies focused on the mechanisms of their beneficial impacts on humans. PT, specifically, has been of particular interest in this research thanks to its bioavailability being 3–4 times higher than resveratrol (80% vs. 20%, respectively), likely due to its single hydroxyl group for metabolic enzymes to act on, with resveratrol having three hydroxyl groups [3,12].

PT is primarily found in blueberries, grapes, peanuts, and the heartwoods of a wide variety of plants [13]. Conveniently, as these are all commonly consumed foods throughout the world, PT is most easily absorbed via oral ingestion, due to its membrane permeability and lipophilicity [14]. In addition to its high bioavailability, PT also has a high degree of cellular uptake and distribution into tissues, contributing to its potential use as a therapeutic agent [13]. In vivo studies have proven that PT distributes to the brain, liver, lungs, kidneys, and heart, all of which are common drug targets [6].

PT has been shown to exhibit an array of anti-inflammatory and antioxidant properties. One of the many mechanisms explored in past research includes its inhibition of inflammation-inducing enzymes like iNOS and COX-2 [15]. Additionally, PT has been shown to potently activate the Nrf2 pathway, which leads to the activation of various downstream antioxidant genes and is used by cells to maintain homeostasis and prevent cancer [13]. These mechanisms used by PT are ways in which this compound displays protective effects for normal human cells against the development of malignancies. The above effects, among others mentioned later in this paper, have been at the center of a growing list of studies into the anticancer properties of this compound.

### 2.2. Pathophysiology of MM

MM is a cancer of the plasma cells of the bone marrow and is currently extremely difficult to treat due to its ability to develop resistance to many of the commonly used treatment regimens. MM is caused by a monoclonal, uncontrolled expansion of a single plasma cell that overwhelms and crowds out other cells that develop within the bone marrow (BM). This overexpansion of the plasma cells in the BM leads to bone pain and sets off a cascade of effects that can present as bone lesions, anemia, hypercalcemia, and renal failure. The majority of these symptoms, originating from bone turnover specifically, have been attributed to downstream effects of disordered osteoclasts (OCs) and osteoblasts (OBs) in the bones of patients with MM. A hallmark of MM bone disease is large, over-abundant OCs with an accompanying decrease in normal OB activity [16]. This combination leads to a high turnover of the bone matrix by OCs without sufficient repair or rebuilding by OBs, which presents as the osteolytic “punched-out” lesions classically associated with MM.

MM is preceded by MGUS, which is defined by the presence of a monoclonal protein without any signs of end organ damage, as is seen in MM [17]. MGUS itself does not require treatment of any kind, but therapy is initiated upon identification to slow disease progression. The transition from MGUS to MM is marked by the clonal plasma cell percentage in the bone marrow exceeding 10% of BM cells.

To properly treat MM once diagnosed, specific targets must be identified by looking at points of dysregulation in normal plasma cells that lead to MM. Many of these points have been identified in past studies into MM cell function. One key feature of MM cells, as well as cells of other cancers, is their dysregulated protein synthesis and turnover. MM cells produce higher than normal quantities of proteins, mainly nonfunctional immunoglobulins, which, as with excessive build-ups of any intracellular protein, leads to increased stress on the endoplasmic reticulum. A way that these cells manage this increase in stress is through an expansion of their endoplasmic reticulum and an activation of the unfolded protein response pathway [18]. Evidence shows that disrupting the proteasome system responsible for the breakdown of excess dysfunctional proteins increases the level of stress on MM cells and can lead to cell death, providing a target for treatment [18,19].

Furthermore, the deregulation of certain Activator Protein-1 (AP-1) transcription factors (TFs) has been implicated as a potential cause of OB and OC dysregulation in MM cells [16]. The deregulation of the JUN, FOS, ATF, and MAF multigene families has been attributed to direct mutations, genetic alterations from external stimuli within the BM, epigenetic changes, and TF dependency on oncogenic activity [16]. These AP-1 TFs play key roles in physiological processes, such as the differentiation of plasma cells so that functional and effective immunoglobulins can be produced.

Another important mechanism of MM proliferation and survival is its upregulation of PD-L1 expression, induced by the pro-inflammatory cytokines Interleukin-6 (IL-6) and Interferon-gamma (IFN-*γ*) [20]. These cytokines are commonly produced within the MM microenvironment and may represent a positive feedback loop between MM cell expansion and increased production of these inflammatory markers. PD-L1 binds to PD-1 on the surface of immune T-cells, inhibiting one of the body’s key defense mechanisms. MM cells exploit this process by increasing the expression of PD-L1 on their surfaces to evade host immune responses and prolong their own survival and proliferation.

The above mechanisms of MM progression and survival, among many others, offer potential therapeutic targets when treating MM. One of the most exploited mechanisms by past treatments has been that of the proteasome, as briefly mentioned above. Proteasome inhibitors, namely bortezomib, which was the first that was widely used for MM treatment, target the ER stress signals to disrupt the cancer cell’s ability to manage the stress as it overproduces proteins [19]. In addition to treatment with proteasome inhibitors, other traditional treatments have included immunomodulatory medications such as thalidomide and lenalidomide, along with monoclonal antibodies like elotuzumab and daratumumab [17].

### 2.3. Anticancer Properties of Pterostilbene

As mentioned previously, PT has been the focus of a long list of studies into the anti-cancer potential of organic, plant-derived compounds. These studies have identified numerous mechanisms in which PT combats cancerous cells separate from the previously mentioned antioxidant and anti-inflammatory properties it shows in healthy cells.

One of the main ways in which PT can eliminate malignant cells is via direct induction of apoptosis [21,22,23]. Some malignancies extend their lifetime via changes in the distribution of regulatory proteins and management of cellular stress. Researchers have identified multiple routes through which PT can dysregulate the control that cancer cells have over these pathways. In both in vitro and in vivo studies using esophageal cancer cells, PT was found to induce Endoreticulum Stress (ERS) via impacts in cellular calcium distribution and redox effects, which increased the activity of the pro-apoptotic Caspase 3 [21]. Similar mechanisms of ERS led to apoptosis in non-small-cell lung cancer cells, both in vitro and in vivo, as well [19]. Furthermore, it was seen in breast cancer cells in vitro that the main way PT inhibited cellular proliferation was via induction of the mitochondrial apoptotic pathway specifically [24]. This was supported by the same project finding an upregulation of the pro-apoptotic protein, Bax, in a concentration-dependent manner of PT [24]. Conversely, PT was also found to downregulate anti-apoptotic proteins Bcl-2 and Bcl-XL in cervical cancer, as well as Bcl-2 and MCL-1 in ovarian cancer in in vitro studies [14,22].

In conjunction with the ERS-related apoptosis PT has been shown to induce, PT also plays an important role in cell cycle regulation of cancer cells. PT was shown to increase the expression and activation of tumor suppressors p53 and p21 in the G1/S phase transition [14,19]. PT was also found to significantly reduce the expression of cell cycle cyclin proteins, including cyclin D1 in the G1/S transition [16,18] and cyclins B1 and E1 in the S and G2 phases [14]. With these findings, PT may qualify as a potential way to limit and fight malignancies at multiple phases of the cell cycle normally exploited by different cancers. Additionally, STAT3, a TF pathway for some of these cell cycle regulators, was found to be downregulated in colon cancer cells and led to apoptosis of these cells [25]. By affecting cyclins, the famous p53 tumor suppressors, and their regulation via STAT3, PT can counteract many of the key strategies employed by various cancer types.

When it comes to tumor progression and spread in general, PT has displayed ways to minimize growth and metastasis of cancers in a variety of ways. In a phase II clinical trial exposing endometrial cancer cells to PT, key tumor progression mechanisms, such as oxidative phosphorylation, glycolysis, hypoxia, and mTOR signaling, were all significantly suppressed [26]. In an in vitro study on lung cancer cells exposed to PT, “massive” chromosomal lesions were created that induced the deterioration of the malignant cells in the G1 phase via the p53 pathway [27]. Furthermore, in pancreatic ductal adenocarcinoma cells, PT was found to induce S-phase cell cycle arrest, as well as autophagy, to inhibit their proliferation in vitro [23]. Of note, these cells were also classified as Gemcitabine-resistant, providing evidence of PT exploiting certain antineoplastic mechanisms not used or exploited by some traditional chemotherapeutics. Lastly, mice that had Diffuse Large B-Cell Lymphoma tumors were exposed to IV PT and found to have significantly reduced tumor growth and weight [28]. This study established that a major mechanism in PT’s anticancer properties was its action on MAPKs, ERK1/2, and p38MAPK in the cancer cells. Another common way for metastasis to occur is through the upregulation of matrix metalloproteinases (MMPs). These enzymes normally help to degrade collagen, but produced in an uncontrolled manner, they can destroy the protective capsule of collagen that limits in situ malignant cells from progressing to widespread. Specifically with PT, MMP-2 and MMP-9 were found to be downregulated in cervical cancer cells after PT administration [14]. All of the above actions may allow PT to help prevent the metastasis of malignant cells.

Another unique way that PT disrupts cancer cell proliferation involves modulating microRNA expression in the cells. One study showed that, in healthy human subjects given 10 mg/day of PT, miR-193b, a tumor suppressor, was found in significantly higher levels than in those given a placebo [12]. This upregulation of miRNA, among the other mechanisms discussed above, displays the dynamic and expansive effects that PT can have on both healthy and cancerous cells, which offers multiple potential routes for further therapeutic research.

### 2.4. Pterostilbene in Hematologic Malignancies

Hematologic malignancies, like MM, differ in many qualities from the solid malignancies that were the focus of earlier discussion. In MM and other hematologic malignancies, the affected cells are concentrated in the BM. The BM is an area of high amounts of cellular division and survival promotion. These two factors make the BM a breeding ground for malignancy and make it highly susceptible to developing leukemias and lymphomas.

Leukemia, involving the white blood cells of the marrow, comes in many varieties based on the stage/age of the cell and its given lineage (myeloid or lymphoid). As these cells, specifically those in the lymphoid lineage, are directly related to the plasma cells impacted by MM, we looked at PT’s impact on this cancer. In Chronic Lymphocytic Leukemia (CLL) specifically, PT was found to be a potent downregulator of overexpressed proteins HSP70 and HSF1 in vitro [29]. In T-lymphoblastic leukemia cell lines in vitro, PT increased the expression of Fas, a cell surface protein that initiates the extrinsic apoptosis pathway [30]. Fas is effectively downregulated by cancer cells in CLL in order to avoid the immune system, but PT causes it to be upregulated to allow Cytotoxic T-Cell-induced apoptosis. Furthermore, in in vivo mouse models, PT was seen to be effective at killing Chronic Myeloid Leukemia (CML) cells positive for the infamous BCR/ABL translocation mutation commonly treated with a tyrosine kinase inhibitor (TKI) such as imatinib [31]. Resistance commonly arises, and PT was found to still maintain effectiveness in eliminating CML cells that had become unresponsive to traditional TKIs.

In the case of lymphomas, as mentioned briefly earlier, PT has been effective against Diffuse Large B-Cell Lymphoma (DLBCL), which, like CLL, referenced above, shares the same lineage as the MM cells we are focusing on. Interestingly, in contrast to the findings of the CLL study, apoptosis in the cells of DLBCL was found to be upregulated by PT via the intrinsic pathway by way of ROS generation and mitochondrial effects [28]. This differs from the extrinsic apoptosis upregulation seen in CLL and supports the idea that PT offers many methods to control, contain, and eliminate hematologic cancers. Via a similar mechanism, PT was able to induce apoptosis and inhibit growth in vitro in T-Cell Leukemia/Lymphoma, largely considered one of the most aggressive hematologic cancers [32]. By the above mechanisms, PT has proven effective at disrupting malignant cell function in hematologic cancers, including those involving the same cell lineages as MM.

### 2.5. Potential Role of Pterostilbene in MM

**Figure 1:**
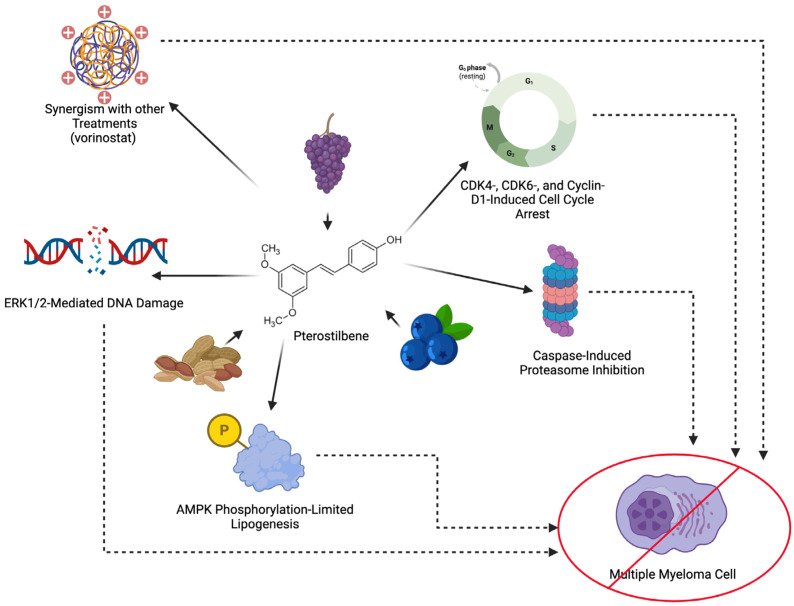
PT comes from a number of natural sources and has been found to exert its anti-cancer properties via multiple mechanisms. These mechanisms align with the ways in which MM cells are able to proliferate and can be exploited in its treatment. Created in BioRender. Hu, J. (2026) https://BioRender.com/8d0by5f (accessed on 14 February 2026).

Having discussed PT and its potential therapeutic application in a wide range of cancers, we now focus on studies that have identified PT’s direct effects on MM (as summarized in Figure 1). MM, known to rapidly develop drug resistance and present recurrently, has a median overall survival of only 6–7 years [16]. As mentioned above, much of the management of cancer development is currently achieved with proteasome inhibitors, namely bortezomib. Currently, “poor-prognostic” patients with a t(14;16) translocation are bortezomib-resistant, characterizing them by a difficulty to effectively treat and manage [16]. Fortunately, one study found PT was able to induce caspase-dependent (intrinsic pathway) apoptosis in vitro in proteasome inhibitor-resistant cells harvested from human MM patients [33]. The same study found that PT may function in S-phase arrest via DNA damage induction in this cell line. Utilizing PT in treatment and management in these patients may help limit the progression of MM while alternative treatment options are explored.

When it comes to inhibiting MM proliferation in non-resistant patients, PT was able to limit energy supplies in malignant plasma cells, as well. In a dose-dependent manner, it was seen that PT increased AMPK phosphorylation and, in turn, limited lipogenesis, a key pathway for energy generation in MM cells [6]. This study was able to display both apoptosis induction in vitro and tumor burden reduction in vivo via this mechanism. Additionally, testing of PT on MM cells in vitro and in vivo showed increased cell cycle arrest in the G0/G1 phase by decreasing CDK4, CDK6, and cyclin-D1, as was found in studies with other cancers [7]. This same study’s findings reaffirmed the value of PT’s pro-apoptotic action via the activation of ERK1/2, a part of the MAPK pathway discussed in other malignancies earlier [7]. Though the overall number of studies specifically on the effects of PT against MM are limited, most of the findings support restricted progression of MM expansion by this natural compound. Ultimately, this calls for future studies to investigate PT’s potential as a survival-extending therapy in MM patients via human trials.

### 2.6. Clinical Relevance

Now that the raw ability of PT to target, inhibit, and treat malignant cells of MM and other cancers has been established, we look to see how this compound has been incorporated into traditional cancer treatment regimens.

Multiple studies have shown that PT expresses a synergistic relationship with different cancer chemotherapies. Specifically, synergism was seen in vitro with the commonly used chemotherapeutic agents cisplatin and 5-Fluorouracil in multiple cell lines of ovarian and colon cancers, respectively [22,34]. One in vitro study using pancreatic cancer cells yielded results supporting PT as an MDR1 modulator that enhances Gemcitabine-induced tumor cell death [23]. In a clinical trial (more details found in Appendix A) with endometrial cancer patients, PT, when combined with Megestrol Acetate, suppressed a number of pro-tumorigenic and inflammatory pathways [26]. Finally, when co-administered with another naturally occurring and plant-derived compound, Thapsigargin, PT was able to significantly decrease viability of esophageal cancer cells via increased ERS levels [21].

Knowing how PT works when administered in conjunction with more traditional therapies in other types of cancer is encouraging, but, in order to assess appropriateness in MM treatment, we first briefly preview current MM treatment strategies. The proteasome inhibitor bortezomib, as mentioned earlier, is traditionally a first line drug among caregivers of MM patients when given in combination with glucocorticoids like dexamethasone. New research on triple combination therapies that add the anti-CD38 antibody daratumumab to the standard bortezomib/dexamethasone regimen have yielded promising results in phase III clinical trials, with a 61.4% lower risk of disease progression or death than in the traditional regimen [35]. However, it should be noted that this addition of daratumumab resulted in a greater incidence of adverse reactions, including Grade 3 and 4 hematologic adverse events [35]. Teclistimab, a T-cell-redirecting antibody, has shown activity that may be superior to some current treatments for heavily pretreated, refractory MM in phase I/II clinical trials [36]. More data regarding sample size and mechanisms can be found in Appendix A. Additionally, CAR T-Cell therapy, a newer modality of cancer treatment, is currently in phase I trials for the treatment of MM [17].

Having reviewed traditional treatment regimens and recent developments, we now look at how PT has been used in conjunction with other MM treatments. In a phase III clinical trial mentioned earlier PT co-administration with the histone deacetylase inhibitor vorinostat to bortezomib-resistant MM cells showed PT demonstrated synergism with increased toxicity to MM cells [35]. This finding, combined with the effects of PT on other chemotherapeutic agents mentioned above, shows that there is potential for PT to be given in conjunction with other agents in the management of refractory MM to yield greater control of the cancer.

The pharmacokinetics of PT have been studied to assess its action and ability as a potential nutraceutical in living models. As mentioned earlier, PT has a higher bioavailability than resveratrol when consumed orally [3]. PT was found to have a superior overall pharmacokinetic profile to resveratrol when compared in animal models [37]. This was mainly attributable to the fact that resveratrol was cleared and eliminated at a significantly higher rate than PT. That being said, PT is still considered to have overall poor bioavailability, and improving its solubility and stability is an aim of investigators. In terms of potential drug interactions, PT has been found to be an inhibitor of the CYP2C8 and UGT1A6 drug-metabolizing enzymes in vitro [38]. Notably, these enzymes metabolize pioglitazone/amodiaquine and serotonin, respectively. This effect must be considered, as the stability of PT is improved with future development.

The safety of PT has been thoroughly studied over the years in various settings, in order to assess potential side effects. In trials on rats, assessing the toxicokinetics of oral PT, minimal subacute toxicity was found [39]. More importantly, a phase II clinical trial investigating the effect of oral PT administration on women with endometrial cancer found only one adverse event in a study of over 30 patients [26]. Another study mentioned earlier, conducted on 30 healthy adult men, found no adverse events during a 12-week course of 100 mg/day of PT [12]. Once a therapeutic dose is established when it comes to the management and treatment of MM, these findings can be truly evaluated for utility in this setting, but, for now, they provide a good basis for safe use of PT in humans at doses much higher than is normally consumed via intake of natural sources.

### 2.7. Future Directives

The documented impact PT has been found to have on cancer cells has shown PT could be a potential supplementary treatment as a nutraceutical for malignancies including MM [40]. The lack of human trials in the context of cancer, and specifically MM, warrants a need for more investigation into dosing and long-term safety. In vitro and in vivo studies have shown what it takes to successfully limit MM cell proliferation with PT. However, what this would look like in large-scale human trials as an oral medication is yet to be seen. Clinical trials must be conducted with PT supplementation in patients with MM diagnoses to evaluate and identify proper dosing that is safe and can still elicit a beneficial effect.

Furthermore, a goal of future research should be to optimize the natural compound in order to enhance the intended pharmacologic impact. Already-existent examples of this are the molecules known as DCZ0825, DCZ0801, and DCZ0805 [8,9,10,11]. These molecules were developed from PT using computer-aided drug design, toxicology, and pharmaceutical chemistry [10]. Testing with these novel compounds was found to have dual-action antitumor activity via apoptosis induction in MM cell lines, as well as conversion of immunosuppressant M2 macrophages to pro-inflammatory M1 macrophages to enhance clearance of cancerous cells [9,10]. This breakthrough in PT-derived compound use for MM cell inhibition shows great potential for medication development. Additionally, these compounds exploit pathways not normally targeted by other MM treatment regimens, which could indicate use as an adjunct treatment with traditional chemotherapeutics. An aim of these studies should be to increase stability of PT-related compounds and, as stated earlier, establish appropriate dosages for human subjects.

## Data Availability

No new data were created or analyzed in this study. Data sharing is not applicable to this article.

## Abbreviations

The following abbreviations are used in this manuscript:

PT	Pterostilbene
MM	Multiple Myeloma
MGUS	Monoclonal Gammopathy of Undetermined Significance
CDK	Cyclin-Dependent Kinase
BM	Bone Marrow
OC	Osteoclast
OB	Osteoblast
AP-1	Activator Protein-1
TF	Transcription Factor
ERS	Endoreticulum Stress
MMP	Matrix Metalloproteinase
CLL	Chronic Lymphocytic Leukemia
CML	Chronic Myeloid Leukemia
TKI	Tyrosine Kinase Inhibitor
DLBCL	Diffuse Large B-Cell Lymphoma

## Appendix A

**Table A1:** Data regarding clinical trials cited in this review using PT as a treatment in hematological malignancies.

Author, Year	Study Design	Population (sample size, *N*)	Malignancy Being Tested	Comparator	Key Finding or Mechanism Explored
Senguttuvan RN et al., 2025 [26]	Phase II randomized window-of-opportunity trial	Endometrial cancer patients (*N* ≈ 40)	Endometrial cancer	Megestrol Acetate alone	Pterostilbene enhances anti-tumor metabolic and apoptotic signaling, improving biological response markers
Palumbo A et al., 2016 [35]	Phase III randomized controlled trial	Relapsed/refractory MM patients (*N* = 498)	Multiple Myeloma	Bortezomib and dexamethasone (Vd)	Addition of daratumumab (anti-CD38 monoclonal antibody) to Vd significantly improved progression-free survival, response depth (CR/MRD negativity), and overall response rate via immune-mediated plasma cell killing
Moreau P et al., 2022 [36]	Phase I/II open-label multicenter trial	Heavily pretreated Relapsed/Refractory MM patients (*N* = 165)	Multiple Myeloma	None (single-arm)	Teclistamab (BCMA × CD3 bispecific antibody) redirects T-cells to MM cells, producing high response rates in triple-class-exposed disease; toxicity dominated by cytokine release syndrome and infections
